# Cholera and Electricity

**Published:** 1849-09

**Authors:** 


### ELECTRICITY AND CHOLERA.

M. Audrand has communicated to the French Academy of Sciences the result of a series of observations, made by him, on the electrical state of the atmosphere during the late prevalence of cholera at Paris; from which he concludes that the epidemic is caused by a deficiency of electricity. He remarked that a large electrical machine, which threw out brilliant sparks in quick succession, when the atmosphere was in a favorable state, appeared to be powerless during the reign of the epidemic. All this time, he adds, there were no thunder-storms. At length, on the evening of the 8th of June, "a storm announced to Paris that electricity had re-entered its domain;" his electrical machine began to give out sparks again, and "in his view cholera was vanishing with the cause that produced it."

In Lexington, we were engaged in a course of lectures on electricity at the time when cholera broke out in that city, and were struck by the fact here noted by M. Audrand. We were unsuccessful in our attempts to excite the fluid in a very powerful machine, and the fact was recorded in the Transylvania Journal of that summer (1833). But in one most important particular our experience differs from that of M. A. Thunderstorms were frequent about the time when we found the machine most inefficient, and recurred every few days, and, more than once, two or three times in a single night, during the first days of the epidemic. That cholera often takes its rise in a peculiar state of the atmosphere—a state characterized by the absence of electricity, appears to be well established, but when once it has gained foothold in a place, it seems to set at defiance every state of weather, pursuing its uninterrupted march through sunshine and storm; when the barometer is high and when it is low; in a humid and sultry, and in a dry and serene atmosphere. And having run its course, without reference to any law of which we have any knowledge, without any sensible change in the air, it begins to decline, and finally disappears. Indeed, up to the present time, we know little of the cause of cholera.

